# HMGB1 level in cerebrospinal fluid as a complimentary biomarker for the diagnosis of tuberculous meningitis

**DOI:** 10.1186/s40064-016-3478-5

**Published:** 2016-10-12

**Authors:** Yan Chen, Jun Zhang, Xiaofei Wang, Yu Wu, Li Zhu, Longkun Lu, Qian Shen, Yanghua Qin

**Affiliations:** 1 Department of Clinical Laboratory Medicine, Shanghai Tenth People’s Hospital, Tongji University, Shanghai, 200072 People’s Republic of China; 2 Department of Laboratory Diagnosis, Changhai Hospital, the Second Military Medical University, Shanghai, 200433 People’s Republic of China; 3 Department of Clinical Laboratory Medicine, Shanghai Pulmonary Hospital, Tongji University, Shanghai, 200433 People’s Republic of China

**Keywords:** HMGB1, Tuberculous meningitis, Biomarker, Cerebrospinal fluid

## Abstract

**Purpose:**

High mobility group box-1 (HMGB1) is a proinflammatory, DAMP protein that participates in many pathological conditions. In this study, we evaluated the usability of CSF HMGB1 as a biomarker for the diagnosis of tuberculous meningitis (TBM).

**Methods:**

A total of 59 TBM patients and 169 control patients were included in our study. CSF samples were obtained and analyzed for HMGB1 using a commercial ELISA kit.

**Results:**

The mean CSF HMGB1 was 19.36 ng/ml in TBM patients (n = 59) versus 3.12 ng/ml in non-TB meningitis patients (n = 30), 2.13 ng/ml in patients with extra neural tuberculosis (n = 73), and 1.06 ng/m in controls (n = 66). According to the receiver operator characteristic curves, a cut-off value of 3.4 ng/ml was calculated, indicating that the sensitivity and specificity of CSF HMGB1 alone in diagnosis of TBM were 61.02 and 89.94 %, respectively. In patients with extra neural tuberculosis and a high risk of TBM, CSF HMGB1 seemed to be a good candidate for early differential diagnosis of TBM at the cut-off value of 3.8 ng/ml, when the sensitivity and specificity were 79.49 and 94.52 % respectively.

**Conclusion:**

Our finding may prove to be clinically useful, because CSF HMGB1 ELISA can be performed in almost all clinical laboratories, especially when sophisticated technologies are either time consuming or unavailable.

## Background

Tuberculous meningitis (TBM), an infectious disease of the central nervous system (CNS), has been on the rise in recent years worldwide, especially in developing countries (Mastroianni et al. 1997). About 30 % of TBM patients die despite anti-TB chemotherapy, mainly due to delayed diagnosis or treatment (Thwaites et al. 2000). The clinical diagnosis of TBM is often difficult because of the complexity of the clinical manifestations and difficulty in differentiating TBM (Kashyap et al. 2001). Currently, the diagnosis of TBM is mainly based on cerebrospinal fluid (CSF) analysis, but both sensitivity and specificity are low, and it is time consuming. It is therefore necessary to find a more sensitive and specific biomarker for quick diagnosis or exclude TBM.

High mobility group box-1(HMGB1) is non-histone nuclear DNA-binding protein ubiquitously expressed in all mammalian cells that plays a vital role in the nuclei of all eukaryotic cells (Lotze and Tracey 2005). HMGB1 protein is considered to be a damage-associated molecular patterns (DAMP) signal in inflammatory conditions (He et al. 2013; Qin et al. 2009, 2014; Scaffidi et al. 2002), tumors (Chung et al. 2009; Lee et al. 2012), and nutritional deficiencies (Ruan et al. 2014; Rickenbacher et al. 2014). Many studies have demonstrated that HMGB1 is a candidate diagnostic marker in diseases such as rheumatoid arthritis (Schierbeck et al. 2013; Kyostio-Moore et al. 2011), inflammatory bowel disease (Palone et al. 2014; Vitali et al. 2011), and sleep apnea syndrome (Wu et al. 2010). So it is reasonable to speculate that HMGB1 level in CSF will increase when CNS was invaded by *M. tuberculosis*.

In the present study, we hypothesize that CSF HMGB1 levels could be a good biomarker to aid in TBM diagnosis, as well as a good candidate to aid in differential diagnosis among TBM high-risk patients. The aim of this study was therefore to evaluate the diagnostic value, sensitivity, and specificity of CSF HMGB1 in TBM patients.

## Methods

### Ethics statement

Our research was approved by the Research Ethics Committee of ChangHai Hospital and Shanghai Pulmonary Disease Hospital. All participants gave informed written consent. All procedures were carried out in accordance with Ethical Guidelines for Biomedical Research Involving Human Subjects of Changhai Hospital. All the data in this study were anonymised.

### Study participants

Our study involved 228 patients who were admitted in ChangHai Hospital or Shanghai Pulmonary Disease Hospital between January 2011 and December 2012. The patients fell into four groups: TBM group (*n* = 59), non-TB meningitis group (meningitis caused by other pathogens) (*n* = 30), extra neural tuberculosis group (pulmonary, intestinal, or joint tuberculosis) (*n* = 73), and control group (*n* = 66).

### Diagnostic criteria of TBM

The diagnostic criteria for TBM were according to Ahuja et al. (1994) as follows:

A. Clinical manifestations: Fever and headache lasting for more than 14 days (mandatory), in addition to vomiting, altered sensorium or the presence of focal deficits (optional).
B. CSF: Pleocytosis with more than 20 cells; predominantly (greater than 60 %) lymphocytes; protein greater than 1 g/l; sugar less than 60 % of the corresponding blood sugar; and negative India ink study and cytology for malignant cells (in relevant situations).
C. Radiological findings: head CT scan showing two or more of the following: ① exudates in basal cisterns or Sylvian fissures; ② hydrocephalus; ③ infarcts; and ④ gyral enhancement.
D. Extra neural tuberculosis: Active tuberculosis of the lung, gastrointestinal tract, urogenital tract, lymph nodes, skeletal system or skin as evidenced by appropriate radiological or microbiological tests or by the presence of caseation necrosis on histopathological examination.

The 4 sub-criteria described above were incorporated into the four groups in a descending order of sensitivity in the diagnosis of TBM.

1. Definite TBM
  (i) Clinical criteria (A)
  (ii) Bacterial isolation from CSF (smear, culture, or TB-PCR) or diagnosis at autopsy
2. Highly probable TBM
  (i) Clinical criteria (A)
  (ii) All 3 of (B) and (C) and (D)
3. Probable TBM
  (i) Clinical criteria (A)
  (ii) Any 2 of B, C and D
4. Possible TBM
  (i) Clinical criteria (A)
  (ii) Any one of (B) (C) and (D)

In this study, definite, highly probable, and probable TBM were considered as TBM, while possible TBM were considered as non-TB meningitis or extra neural tuberculosis.

### CSF processing

CSF samples were centrifuged immediately at 1300×*g* for 10 min, and the supernatant was stored at −80 °C in a 1.5 ml microcentrifuge tube prior to analysis for HMGB1.

### Measurement of CSF HMGB1 levels

The concentration of HMGB1 was measured by the commercially available HMGB1 ELISA Kit II (SHINO-TEST Corporations, Kanagawa, Japan) according to the manufacturer’s instructions. Briefly, 100 μl sample diluent was added to each well, and then 10 μl standard, sample or control was added to the well. The microtiter plate was incubated for 20–24 h at 37 °C. After washing, 100 μl/well of anti-humanHMGB1 peroxidase-conjugated monoclonal antibody was added and the plate was incubated at room temperature for 2 h. After washing, the chromogen 3,3,5,5′-tetra-methylbenzidine was added to each well. The enzyme reaction was allowed to proceed for 30 min at room temperature. The chromogenic substrate reaction was terminated by addition of the stop solution (0.35 mol/l Na_2_SO_4_), and the absorbance was read at 450 nm. The results were calculated using a calibration curve prepared from standards.

### Western blotting of CSF HMGB1

A total volume of 30 μl cell free CSF sample was boiled with SDS loading buffer containing protease inhibitor, separated by SDS-polyacrylamide gel electrophoresis (SDS-PAGE), and then transferred to the nitrocellulose membrane (Millipore, Darmstadt, Germany). Blots were blocked by 5 % defatted milk for 2 h, incubated with primary HMGB1 antibody (1:500, SaierBio, Tianjin, China), and further incubated with goat anti-rabbit HRP-conjugated secondary antibody (1:2000, Cell Signaling, Beverly, MA). The membrane was visualized with an ECL Western Blotting System (Pierce Protein Research Products, Rockford, IL, USA).

### Statistical analysis

Statistical analysis was performed by Graphpad Prism 5.0 (Version 5.0, La Jolla, CA, USA). To compare the concentrations of HMGB1 in patients and controls, *p* values were calculated for the average ELISA results by one-way ANONA test and Newman-Keuls Multiple Comparison Test. The ROC curve was constructed for CSF HMGB1, by which the sensitivity, specificity, error rate and AUC were calculated to determine the diagnostic accuracy of our findings.

## Results

### Patient demographic data

The male/female in the TBM, non-TB meningitis, extra neural tuberculosis and control groups was 33/26, 21/9, 40/33, and 46/20, respectively. In non-TB meningitis group, there are 16 viral meningitis, 9 bacterial meningitis, and 5 cryptococcal meningitis; in extra neural tuberculosis group, there are 68 pulmonary tuberculosis, 3 intestinal tuberculosis, and 2 joint tuberculosis. Patient data including age, sex and the mean level of HMGB1 are shown in Table 1.

**Table 1:** CSF HMGB1 levels in patients with TBM, non-TB meningitis and extra neural tuberculosis, and in control subjects

	TBM (n = 59)	Non-TB meningitis (n = 30)	Extra neural tuberculosis (n = 73)	Control (n = 66)
Sex (M/F)	33/26	21/9	40/33	46/20
Age (mean, range)	32.61 (14–90)	31.09 (8 m–59)	37.05 (2–80)	47.82 (7–79)
HMGB-1 ($\bar{x} \pm {\text{sd}},{\text{ng}}/{\text{ml}}$)	19.36 ± 4.75	3.12 ± 2.00	2.13 ± 0.48	1.06 ± 2.17

Group1: TBM patients; group 2: non-TB meningitis; group 3: Extra neural tuberculosis; group 4: control group

*M* male, *F* female, *HMGB1* high mobility group box protein-1

### HMGB1 level in diseased people and controls

**Fig. 1:**
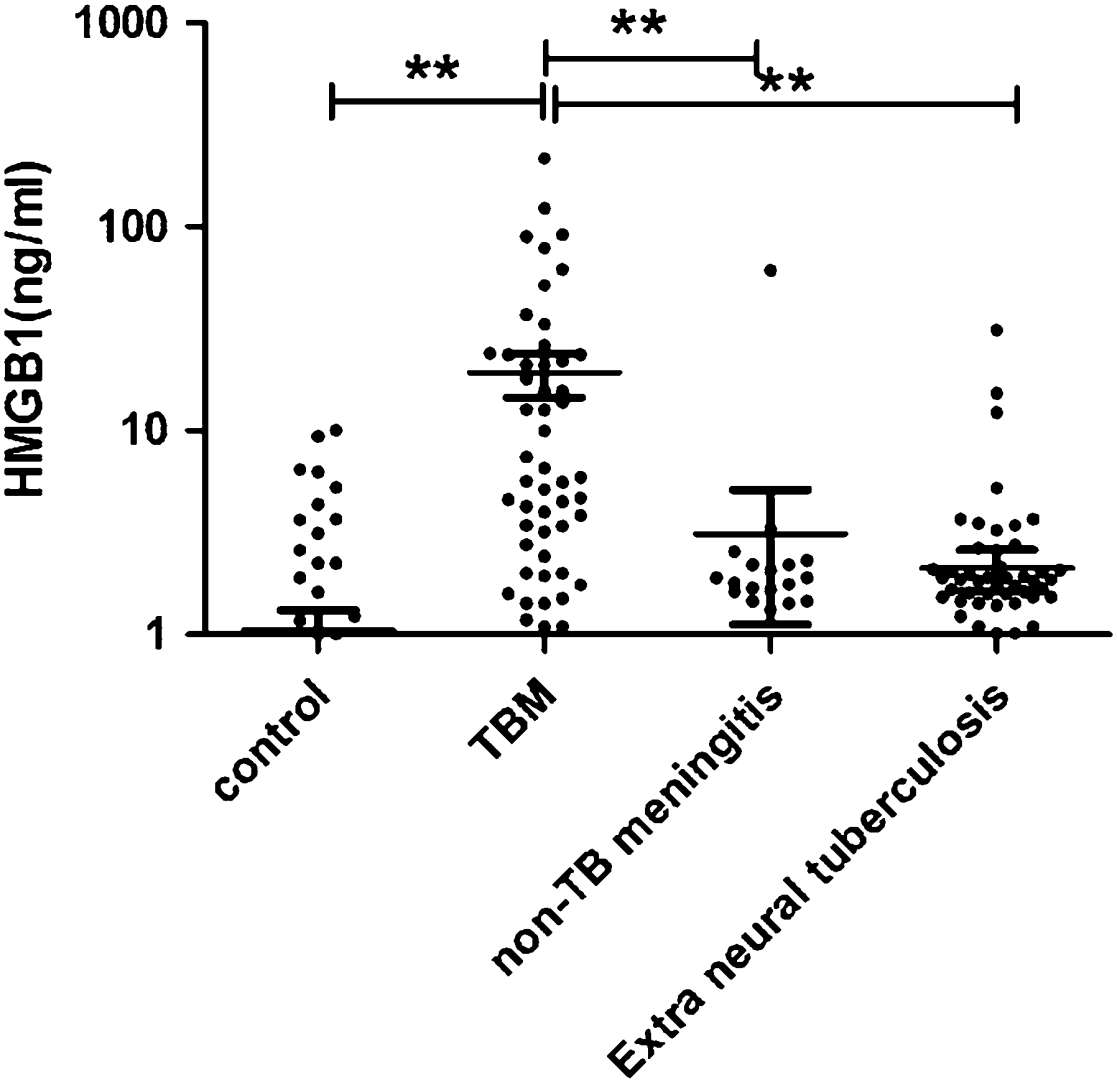
Dot plots of CSF HMGB1 in TBM, non-TB meningitis, extra neural tuberculosis, and control groups. The mean CSF HMGB1 was significantly higher in TBM group than others (*p* < 0.001)

**Fig. 2:**
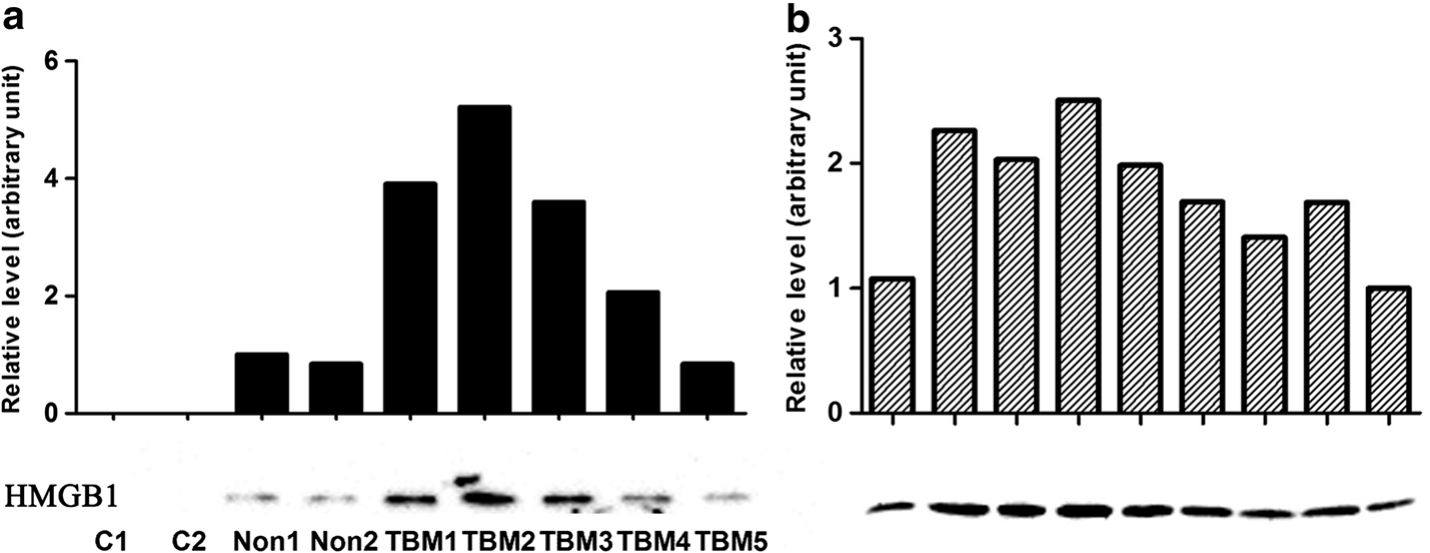
Western blotting and grayscale analysis of CSF HMGB1. **a** Samples from 2 controls, 2 no-TBM patients and 5 TBM patients; **b** Samples from 9 definite TBM patients. 30 μl CSF was loaded and separated by SDS-PAGE, and HMGB1 was detected using a monoclonal anti-HMGB1 antibody. The *grayscale value* of protein bands were analyzed by ImageJ software

The HMGB1 concentration was measured by ELISA, and results are shown in Fig. 1. The mean HMGB1 value in TMB group, non-TB meningitis, extra neural tuberculosis, and control groups was 19.36, 3.12, 2.13, and 1.06 ng/ml, respectively, showing a significant difference among groups (*p* < 0.001). Subgroup comparison CSF HMGB1 levels in TBM patients were significant higher than those in the other groups (*p* < 0.001), and there was no significant difference between non-TB meningitis, extra neural tuberculosis and control groups (*p* > 0.05). To confirm the ELISA result, CSF samples from 5 TMB patients, 2 non-TB meningitis patients and 2 people were analyzed using western blotting. The results are shown in Fig. 2a, indicating that the intensity of the bands in TMB patients was higher than that in the others groups. Above all, there were 9 definite TBM cases in our study, and the clinical data and western blotting result were shown in Table 2 and Fig. 2b. The concentration of CSF HMGB1 in 9 definite TBM patients (37.50 ng/ml) was significant higher than the mean level of CSF HMGB1 in TBM group.

**Table 2:** Clinical data in definite TBM patients

Case no.	Year	Sex	Complains	Lab outcome	HMGB1 (pg/ml)
41	34	F	Cough, fever for 1 month	PCR	19.96
48	20	M	Headache, dizziness for 1 month, with mild nuchal rigidity	PCR	68.80
51	21	F	Fever, chest pain for 2 months, with nuchal rigidity	Acid-fast staining and PCR	51.71
60	21	F	Headache for 1 month	PCR	79.32
97	48	M	Treated for pulmonary tuberculosis for 7 months, complained for intermittent headache and malaise for 1 month	Culture	31.08
113	20	M	Treated for pulmonary tuberculosis for 7 months	Culture	23.96
132	24	F	Fever for 2 months, and brain MRI shows multiple cerebellar tuberculoma	PCR	21.06
140	14	F	Treated for pulmonary tuberculosis for 8 months, and brain MRI shows multiple tuberculoma	Culture	23.71
176	20	M	Treated for pulmonary tuberculosis for 10 months, and brain MRI shows vascular lesions	PCR	17.92

### Evaluation of the sensitivity and specificity of HMGB1 for the diagnosis of TBM

**Fig. 3:**
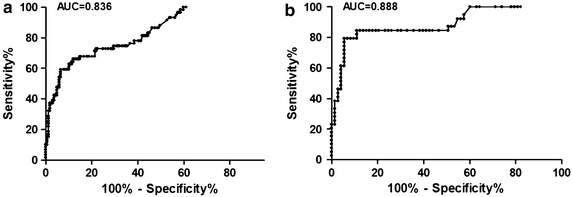
ROC curve of CSF HMGB1 showing the threshold value of CSF HMGB1 in the diagnosis of TBM. **a** CSF HMGB1 reached a sensitivity of 61.02 %, a specificity of 89.94 %, a positive predictive value of 67.92 %, and a negative predictive value of 86.86 % (cut-off value: 3.4 ng/ml; area under the curve: 0.836); **b** In tuberculosis patients, CSF HMGB1 reached a sensitivity of 79.49 %, a specificity of 94.52 %, a positive predictive value of 88.57 %, and a negative predictive value of 89.61 % (cut-off value: 3.8 ng/ml; area under the curve: 0.888)

The receiver operator characteristic (ROC) was evaluated with respect to sensitivity and specificity. The area under the curve (AUC) was 0.836. For the diagnosis of TMB, the best cut-off point for HMGB1 was 3.4 ng/ml, and the calculated sensitivity and specificity were 61.02 % (95 % CI 47.44–73.45 %) and 89.94 % (95 % CI 84.38–94.03 %), respectively (Fig. 3a).

Further investigation was performed to see whether CSF HMGB1 level could be used as a biomarker to help determine whether or not the CNS was involved in extra neural tuberculosis. As showed in Fig. 3b, at a cut-off of 3.8 ng/ml, the calculated sensitivity and specificity were 79.49 % (95 % CI 63.54–90.70 %) and 94.52 % (95 % CI 86.56–98.49 %), respectively. The positive and negative predictive value was 88.57 % (31/35) and 89.61 %, respectively.

## Discussion

TBM infection remains a global health problem. According to the World Health Organization (WHO) report (WHO 2003), the incidence of TBM is increasing by 0.4 % every year worldwide, especially in developing countries. TB infection is also the major reason of morbidity and mortality. Delayed or no treatment will increase the risk of developing irreversible neurologic sequelae and death. The clinical diagnosis of TBM is often difficult due to the pleomorphic clinical presentation. Routine CSF-biochemical analysis including pleocytosis with lymphocytosis may increase protein levels and reduce glucose levels (Thwaites et al. 2000). Despite the multiple modern diagnostic tools currently available, the diagnosis of TBM still depends primarily on the patient history and clinical findings. The confirmed diagnosis of TBM depends on the detection of *M. tuberculosis* from the CSF, and careful and repeated search for acid fast bacilli (AFB) by Ziehl-Neelsen staining or culture. Although these two methods are considered the gold standard for the diagnosis of TBM, the resultant yield via direct CSF staining for AFB is as low as 10–20 % (Bloom 1994). In addition, culture of *M. tuberculosis* via solid media such as Lowenstein-Jensen usually takes 8 weeks, often with a negative result (Thwaites et al. 2002). Mycobacterial Growth Indicator Tube (MGIT) is a liquid culture system developed by Becton–Dickinson (BD, Franklin, NJ,USA), and has a better sensitivity for diagnosis of TBM, but it requires special culture and detection equipment and still needs at least 3–4 weeks (Hannan et al. 2010; Thakur et al. 2010). Newer nucleic acid amplification techniques (NAAT) such as polymerase chain reaction (PCR) developed for the detection of *M. tuberculosis* in clinical specimens are more sensitive and specific but not available for widespread use in developing countries. The Xpert^®^ MTB/RIF test (Cepheid, Sunnyvale, CA, USA) is an automated, cartridge-based PCR test used to detect *M. tuberculosis* in sputum, and the World Health Organization (WHO) recommends it as initial TBM diagnostic test. With proper sample processing, such as centrifugation, Xpert reaches a sensitivity as high as 70–80 % (Bahr et al. 2015; Patel et al. 2013), and can detect rifampin resistance the same time. The shortcoming of Xpert is obvious since this test and concessional price ($9.98) are not widely available (Puri et al. 2016). So, a rapid, easily applied and inexpensive diagnostic tool with a high diagnostic value for TMB is badly needed.

HMGB1 is not a new protein. It was initially named for its characteristic rapid electrophoretic mobility in polyacrylamide gels (Harris and Raucci 2006), as a late-phase mediator of inflammation is induced by early proinflammatory cytokines. Knowing that CSF proteins can reflect the state of the CNS, accurate analysis of CSF should be able to provide some valuable information about the neurological health of a patient. As HMGB1 can be actively secreted from immune cells or passively released from dying cells into extracellular space (Lotze and Tracey 2005), the release of HMGB1, which functions as a danger signal, represents a stress condition and inflammatory response to certain stimuli (Chung et al. 2009). A prospective observational study by Goldstein et al. (2006) showed that serum HMGB1 levels were elevated in patients with cerebral vascular ischemia. A recent study found that HMGB1 could be detected in the CSF of encephalitic dogs. We conclude that HMGB1 is present in the CSF of people suffering from TMB.

In this study, we investigated CSF HMGB1 levels in TB patients and evaluated the potential value of HMGB1 as a diagnostic biomarker. Our results showed that CSF HMGB1 levels in TBM patients were elevated markedly as compared with those in the other control groups, especially in control subjects in whom the expression of HMGB1 was very low or even undetectable, indicating the HMGB1 is an ideal test biomarker to reflect the inflammatory status of CNS. As a DAMP, CSF HMGB1 level may increase under certain conditions such as inflammation and ischemia. Our results suggested that the best cut-off value point was 3.4 ng/ml for the diagnosis of TBM, when the calculated sensitivity and specificity were 61.02 and 89.94 %, respectively. Although CSF HMGB1 levels between TBM patients and control patients differed significantly, a high discriminative ability was not obtained in the curve. According to by Albayrak et al. (2011), whether or not the serum level of HMGB1 could be used as a diagnostic biomarker for the diagnosis of acute appendicitis was merely another blind alley that remained to be seen according to the result of ROC, which is similar to the situation in our study, where the sensitivity was not sensitive enough while the specificity was very good according to the result of HMGB1. However, because the confirmation of the diagnosis of TBM is very difficult and CSF HMGB1 would be helpful to identify TBM when direct smear is negative, PCR test isn’t available, and waiting for culture result. We further investigated whether CSF HMGB1 level was a good biomarker to identify TBM in patients who suffered from extra neural tuberculosis such as pulmonary tuberculosis, intestinal tuberculosis, or joint tuberculosis. The sensitivity and specificity were 79.49 % (31/39) and 94.52 % (69/73) respectively at a cut-off value of 3.8 ng/ml. Our results seem to suggest that CSF HMGB1 level is a good diagnostic biomarker for patients with extra neural tuberculosis who have a high risk of TBM. If so, the time needed for the diagnosis of TBM would be much shorter and patients could receive treatment earlier. In TBM group, CSF HMGB1 in 9 definite TBM patients (37.50 ng/ml) was significant higher than highly probable, and probable TBM patients means some of the clinical diagnosis TBM may not accurate. Since CSF HMGB1 has high specificity, it may be a useful tool in helping physicians to rule out TBM.

## Conclusion

In conclusion, our results showed that HMGB1 levels in the CSF of TBM patients were significantly higher than those in control group, non-TB meningitis, or extra neural tuberculosis. In addition, HMGB1 may be a helpful candidate biomarker to differentiate TBM from TB in earlier stages, predict disease prognosis, or at least confirm neurological disorders in confirmed cases of TBM. However, further investigations in larger cohorts of individuals are needed to confirm the relevance and usefulness of HMGB1 as an early diagnostic marker for monitoring patients with a high risk of TBM. The possibility to use HMGB1 as an early diagnosis biomarker for the detection of TB-infection in the CNS could avoid unnecessarily treatment and improve the outcome and prognosis of TBM patients.
